# Collaborative Referral Model for Hepatitis C Screening and Treatment in a Remote Mountainous Region of Taiwan during the COVID-19 Pandemic

**DOI:** 10.3390/v15040827

**Published:** 2023-03-24

**Authors:** Chi-Ming Tai, Ming-Jong Bair, Tzu-Haw Chen, Cheng-Hao Tseng, Chih-Cheng Chen, Hung Lam, Ming-Lung Yu

**Affiliations:** 1 Division of Gastroenterology and Hepatology, Department of Internal Medicine, E-Da Hospital, I-Shou University, Kaohsiung 824, Taiwan; chimingtai@gmail.com (C.-M.T.);; 2 School of Medicine for International Students, College of Medicine, I-Shou University, Kaohsiung 824, Taiwan; 3 Division of Gastroenterology, Department of Internal Medicine, Taitung Mackay Memorial Hospital, Taitung 950408, Taiwan; 4 Department of Medicine, Mackay Medical College, New Taipei 252, Taiwan; 5 Division of Gastroenterology and Hepatology, Department of Internal Medicine, E-Da Cancer Hospital, I-Shou University, Kaohsiung 824, Taiwan; 6 School of Medicine, College of Medicine, I-Shou University, Kaohsiung 824, Taiwan; 7 Liouguei District Public Health Center, Kaohsiung 844, Taiwan; 8 School of Medicine, College of Medicine and Center of Excellence for Metabolic Associated Fatty Liver Disease, National Sun Yat-sen University, Kaohsiung 804, Taiwan; 9 Hepatobiliary Division, Department of Internal Medicine, Kaohsiung Medical University Hospital, College of Medicine, Kaohsiung Medical University, Kaohsiung 807, Taiwan; 10 Division of Hepato-Gastroenterology, Department of Internal Medicine, Kaohsiung Chang Gung Memorial Hospital, Kaohsiung 833, Taiwan

**Keywords:** direct-acting antiviral, viral hepatitis, hepatitis C, primary care, referral model, screening, elimination

## Abstract

Community-based screening for the hepatitis C virus (HCV) decreased during the COVID-19 pandemic. We developed a collaborative referral model between a primary clinic (Liouguei District Public Health Center, LDPHC) and a tertiary referral center to increase HCV screening and treatment uptake in a mountainous region of Taiwan. Once-in-a-lifetime hepatitis B and C screening services established by the Taiwan National Health Insurance were performed at LDPHC. Antibody-to-HCV (anti-HCV)-seropositive patients received scheduled referrals and took a shuttle bus to E-Da hospital for HCV RNA testing on their first visit. Direct-acting antiviral agents (DAAs) were prescribed for HCV-viremic patients on their second visit. From October 2020 to September 2022, of 3835 residents eligible for HCV screening in Liouguei District, 1879 (49%) received anti-HCV testing at LDPHC. The overall HCV screening coverage rate increased from 40% before referral to 69.4% after referral. Of the 79 anti-HCV-seropositive patients, 70 (88.6%) were successfully referred. Of the 38 HCV-viremic patients, 35 (92.1%) received DAA therapy, and 32 (91.4%) achieved sustained virological response. The collaborative referral model demonstrates a good model for HCV screening and access to care and treatment in a Taiwan mountainous region, even during the COVID-19 pandemic. Sustained referral is possible using this routine referral model.

## 1. Introduction

Chronic hepatitis C virus (HCV) infection can progress to cirrhosis and hepatocellular carcinoma (HCC), increasing mortality associated with hepatic and extrahepatic disease [1]. Approximately 71 million people were affected by HCV infection in 2015, representing a 1.0% global prevalence [2]. The introduction of direct-acting antiviral agents (DAAs) for treating HCV infection has remarkably improved treatment safety and efficacy [3]. Viremic HCV infections are estimated to have decreased to 56.8 million people at the beginning of 2020 [4]. Given the decreasing trend, the World Health Organization (WHO) has set an ambitious goal to eliminate HCV infection as a public health threat by 2030 [5].

Although the estimated prevalence of HCV infection in Taiwan is 1.8–5.5%, which is much higher than the global prevalence, Taiwan aims to achieve the WHO goal by 2025 [6,7]. Despite the high rates of treatment response and nationwide coverage of insurance reimbursement in Taiwan, a large gap remains between clinical efficacy and community effectiveness in HCV treatment [8]. For special populations, such as people who inject drugs and patients on uremic hemodialysis, various strategies are used to overcome barriers, with good results [9,10]. Community-based screening for HCV infection among residents and outreach clinics for HCV treatment are common approaches for HCV elimination in rural or remote areas of Taiwan, which have been found to be effective [11,12,13]. Kee et al. reported community-based screening for antibodies to HCV (anti-HCV) reflex HCV Ag test in two HCV hyperendemic townships and found that a localized care delivery model using an outreach hepatology clinic is feasible to enhance accessibility for HCV treatment [11]. Tien et al. also reported the effectiveness of village-by-village anti-HCV screening tests and linking to outreach hepatology care at two indigenous townships. They reached a screening coverage of 73.5% in residents, and 74.9% of anti-HCV-seropositive patients visited an outreach hepatology clinic for HCV treatment evaluation [12]. Tsai et al. reported HCV infection was decreasing in an hyperendemic area through continuing education, prevention, and treatment strategies [13]. However, this strategy requires support from medical centers and is usually of limited duration due to the lack of sufficient support. Moreover, screening costs typically increase when HCV prevalence is low [14]. Therefore, tailored screening and referral strategies need to be developed based on different population characteristics. In addition, coronavirus disease 2019 (COVID-19), which became a pandemic in 2020, delayed or canceled many HCV elimination programs [15]. Community-based screening, which largely influenced the provision of HCV screening and treatment services in remote areas, decreased or was even discontinued during the COVID-19 pandemic.

Liouguei District is located in the mountainous region of southern Taiwan where medical resources are scarce, and only three clinics provide medical care in this area. Liouguei District Public Health Center (LDPHC) is the largest primary care clinic in the whole district. During the COVID-19 pandemic, LDPHC not only provided medical services to residents, but was also responsible for public health tasks, such as COVID-19 vaccination. The LDPHC provided us with an opportunity to perform HCV screening at a defined physical facility in the Liouguei area. Therefore, the present study aimed to develop a collaborative referral model between a primary clinic (LDPHC) and a tertiary referral center (E-Da hospital) to increase HCV screening and HCV treatment uptake in a remote mountainous region of Taiwan during the COVID-19 pandemic.

## 2. Materials and Methods

### 2.1. Study Setting and Participants 

Liouguei District is located in an area of 194.2 km^2^ in the mountainous region of Kaohsiung City in southern Taiwan. It had a population of 12,401 residents in October 2020. LDPHC is about 51 km away from E-Da Hospital and the chief of LDPHC is a general practitioner. Since 28 September 2020, the Taiwan National Health Insurance (NHI) has provided once-in-a-lifetime hepatitis B and C screening services for people between the ages of 45 and 79 years, and this service for HCV screening was used as the basis for the collaborative referral model. Among the residents in Liouguei District, 6393 residents were aged between 45 and 79 years. Among the 6393 residents, 2558 residents received anti-HCV testing before October 2020, and the HCV screening coverage rate before this referral model started was 40.0%. After excluding residents who had received prior anti-HCV testing, 3835 (60.0%) residents met the Taiwan NHI criteria for HCV screening. The study protocol was reviewed and approved by the Ethical Committee of E-Da Hospital. 

### 2.2. Collaboration of a Multidisciplinary Team

**Figure 1:**
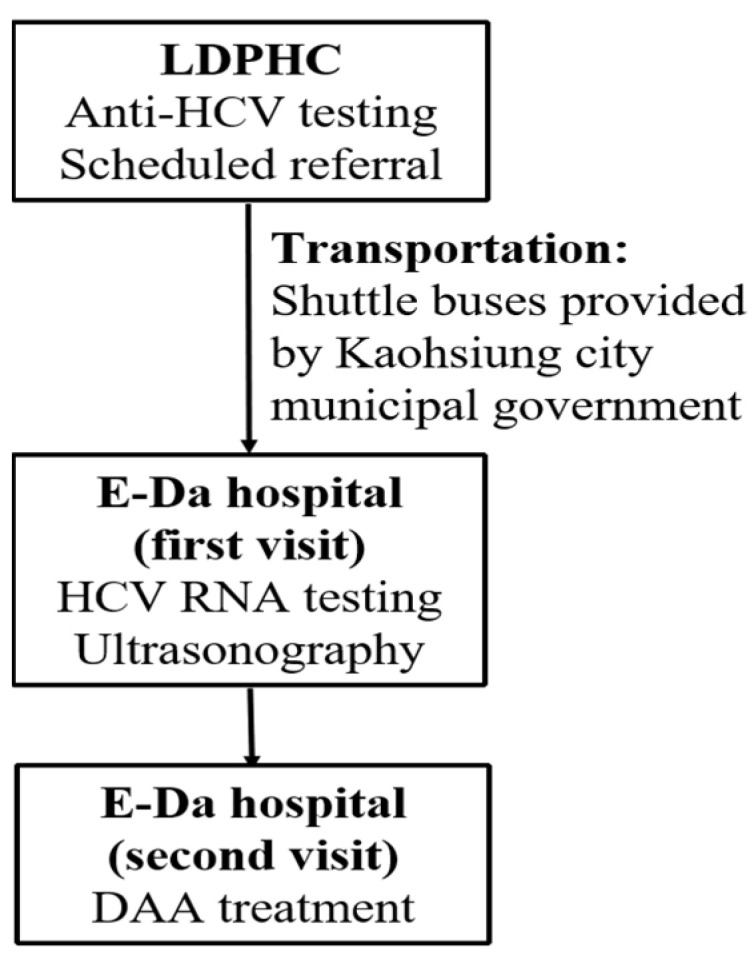
Referral model between LDPHC and E-Da hospital. LDPHC, Liouguei District Public Health Center; DAAs, direct-acting antivirals.

On 1 January 2019, the Taiwan NHI authorized coverage of DAA prescriptions for all HCV-viremic Taiwanese citizens, but such DAA therapy must be prescribed by hepatologists or infectious disease specialists. Therefore, a collaborative care team composed of LDPHC staff and the hepatology department staff of E-Da hospital was established. HCV screening was performed at LDPHC, and anti-HCV-seropositive residents were referred to E-Da hospital for HCV RNA testing, ultrasonography, and DAA treatment (Figure 1). 

The major steps and strategies to overcome barriers were as follows (Table 1).

#### 2.2.1. Anti-HCV Testing at LDPHC 

HCV screening as part of integrated services in the model was performed at LDPHC. LDPHC staff used the in-house electronic system to determine whether or not residents had received prior anti-HCV testing. Then, LDPHC staff attempted to refer all anti-HCV-seropositive patients to E-Da hospital except for those who had records of undetectable HCV RNA or evidence of sustained virological response (SVR) after being treated with pegylated interferon/ribavirin or DAA. All residents were encouraged to receive anti-HCV testing if they had not received prior testing.

#### 2.2.2. Referral to E-Da Hospital 

Transportation is a major barrier that hinders residents in Liouguei District from seeking medical help from a referral hospital. To solve this problem, the Kaohsiung city municipal government provides shuttle buses from the mountainous regions in Kaohsiung to a medical center. The shuttle bus was also used for the referred patients in the present model. Scheduled appointments with a physician who is familiar with this model allows patients to go directly to the physician’s clinic, which increases patients’ acceptance of referral and the overall referral rates.

#### 2.2.3. DAA Treatment and Pre-Treatment Evaluation in E-Da Hospital

Pre-treatment evaluation, including HCV RNA testing and abdominal ultrasonography for detection of cirrhosis and hepatocellular carcinoma (HCC), was performed on referred patients’ first visit to the liver clinic at E-Da hospital. Ultrasonographic signs of cirrhosis include nodular liver surface, coarse echotexture, and splenomegaly. DAAs were prescribed for HCV-viremic patients at the second visit. Simplified processes were adopted to reduce outpatient waiting time for seeing a doctor and receiving ultrasonography, which enhance patients’ acceptance for referral and treatment.

### 2.3. Assessment of Treatment Responses

Glecaprevir/pibrentasvir (GLE/PIB) and sofosbuvir/velpatasvir (SOF/VEL) were the DAAs of choice for HCV treatment in this collaborative model, and the decision was made jointly by the patient and the hepatologist. Complete treatment was defined as completion of the entire treatment regimen, usually 8 weeks and 12 weeks for GLE/PIB and SOF/VEL, respectively. The treatment uptake rate was defined as the proportion of HCV-viremic patients who were treated among the total number of HCV-viremic patients referred. Serum HCV RNA was assessed at the end of treatment and 12 weeks after the cessation of treatment. SVR12 was defined as undetectable HCV RNA 12 weeks after the cessation of treatment. Non-SVR was divided into virologic failure and non-virologic failure (i.e., other explanations for incomplete treatment such as death during treatment or lost to follow-up). For the present study, virologic failure and non-virologic failure were defined as detectable HCV RNA and lack of available HCV RNA data at post-treatment week 12, respectively, as described previously [9].

### 2.4. Statistical Analysis

Descriptive results for continuous variables are presented as mean ± standard deviation (SD), and categorical variables are presented as percentages. Serum HCV RNA level was expressed as the logarithmic transformation of the original values. SVR12 was estimated by intention-to-treat (ITT) analysis (all patients receiving DAAs) and per-protocol (PP) analysis (patients receiving DAA with HCV RNA data available at post-treatment week 12, excluding non-virological failures). Anti-HCV-seropositive patients who were successfully referred were divided into patients with detectable HCV RNA and patients without detectable HCV RNA. Characteristics of these two groups were compared using Student’s *t* test, Chi-square test, or Fisher’s exact test, as appropriate; *p* < 0.05 was established as statistical significance. The commercial statistical software package (version 9.4; SAS Institute, Inc., Cary, NC, USA) was used for all statistical analyses.

## 3. Results

### 3.1. Screening Coverage Rate and Referral Rate 

**Figure 2:**
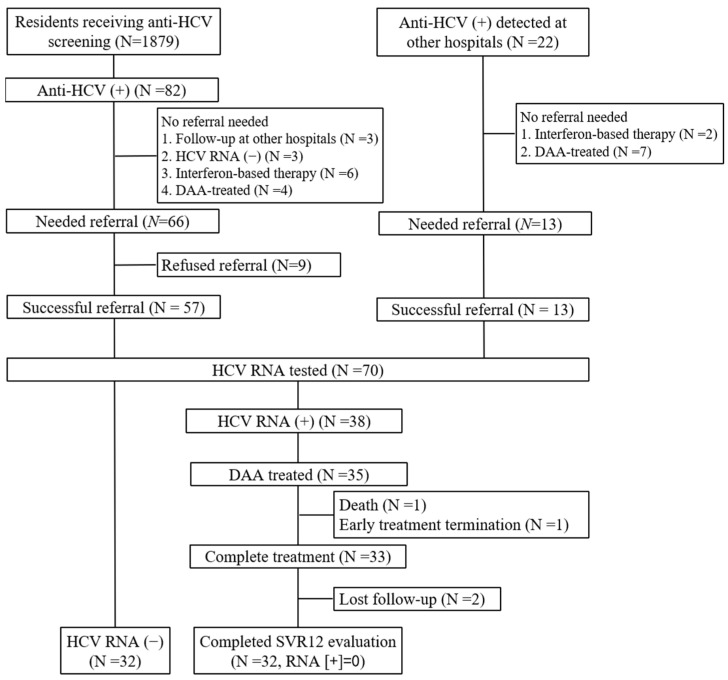
Flowchart of participants in the referral model. DAA, direct-acting antiviral; SVR, sustained virological response.

From October 2020 to September 2022, 1879 out of 3835 residents (49.0%) eligible for Taiwan NHI hepatitis B and C screening received testing for hepatitis B virus surface antigen (HBsAg) and anti-HCV at LDPHC (Figure 2). The overall screening coverage rate of 6393 residents in Liouguei District increased from 40% before referral to 69.4% after referral (*p* < 0.001). A total of 82 residents were HCV-infected, with an anti-HCV seroprevalence of 4.4%. Of the 82 anti-HCV-seropositive patients, 16 did not need referrals, including follow-up at other hospitals (3 patients), HCV RNA-seronegative (3 patients), or successful antiviral therapy (10 patients, 6 with interferon-based therapy and 4 with DAA therapy). Some 22- anti-HCV-seropositive patients received anti-HCV testing at other hospitals, and 13 patients needed referrals. Of 79 anti-HCV-seropositive patients who needed referral, successful referral was achieved in 70 patients (88.6%), and all patients received HCV RNA testing and ultrasonography in E-Da hospital. 

### 3.2. Characteristics of the Anti-HCV-Seropositive Subjects with Successful Referral

The mean age of the 70 patients with successful referral was 64.4 years, and 31 (44.3%) patients were male. Of the sample, 7 patients (10%) were HCV/HBV coinfected, and 3 patients (4.3%) had compensated cirrhosis. Among the 70 patients referred, there were 38 patients (54.3%) with detectable HCV RNA and 32 patients (45.7%) without (Table 2). HCV-viremic patients had significantly higher AST and ALT levels than those without detectable HCV RNA (*p* = 0.005 and 0.003, respectively). Of the 38 HCV-viremic patients, the mean HCV RNA level was 5.9 ± 1.0 log IU/mL, and the major genotypes were genotype 1 and genotype 2. Of note, HCC was found in four patients, and all four were HCV-viremic patients. Two of the four patients had cirrhosis, and none was HBV co-infection. 

### 3.3. Treatment Outcomes of 35 Patients Receiving DAA

Among the 38 HCV-viremic patients, 3 patients did not receive DAA therapy, including 1 with advanced HCC (*n* = 1) and 2 unwilling to receive DAA therapy (*n* = 2) (Figure 2). GLE/PIB and SOF/VEL were prescribed to 28 and 7 patients, respectively. The major reason was the shorter treatment period of GLE/PIB. Of 35 patients who received DAA therapy, 2 did not complete antiviral treatment. One patient died of cirrhosis with septic shock. The other patient discontinued GLE/PIB 4 weeks after treatment because of hyperbilirubinemia (5.48 mg/dL). However, his bilirubin level returned to normal after discontinuation of DAA, and he still achieved SVR12. Finally, 32 out of 35 patients (91.4%) achieved SVR12. All three patients who did not achieve SVR12 were considered non-virologic failures, including one who died during treatment and two who were lost to follow-up. The rates of SVR12 were 91.4% (32/35) and 100% (32/32) in ITT and PP analysis, respectively (Table 3).

## 4. Discussion

Results of the present study confirmed that referral and treatment uptake of anti-HCV-seropositive patients in a Taiwan mountainous region are feasible after a collaboration was established between a primary clinic and a tertiary referral center, even during the COVID-19 pandemic. The successful referral rate and treatment uptake rate were 88.6% and 92.1%, respectively. Aggressive screening strategies, scheduled referral, a shuttle bus direct to E-Da hospital, and simplified processes were key to increasing the success of referral in this newly developed collaboration model for screening and treating HCV in a remote region of Taiwan.

Screening is a critical step on the road toward HCV elimination. Although HCV screening and treatment is challenging in remote areas, studies have demonstrated that outreach through continuous screening programs increased accessibility and cost-effectiveness in community HCV screening programs [12,13]. However, this strategy needs support from medical centers and usually focuses on areas with high HCV prevalence. Therefore, certain remote areas, including Liouguei District, do not receive sufficient support, and a more refined strategy is needed. Primary clinics are the major medical service providers in remote areas and have the most frequent contact with residents. Therefore, it is reasonable to cooperate with primary clinics to build a continuous HCV screening model. LDPHC reached a high screening rate even during the COVID-19 pandemic, and several factors help to explain why. First, the adoption of strategies to enhance uptake of anti-HCV testing, including peer workers, clinician reminders for testing, and provision of testing as part of integrated services as recommended by the WHO 2017 testing guidelines [16]. LDPHC staff incorporated HCV screening into the daily workload and took advantage of any opportunity to educate and encourage all residents who visited the LDPHC for medical needs to receive HCV screening. Using this method, HCV screening became a daily routine that could be continuously performed at LDPHC. Second, LDPHC staff provided medical services and promoted public health to a large proportion of residents in Liouguei District and thereby had the opportunity to contact most of residents in the district. Although the COVID-19 pandemic decreased the demand for medical services in general, it also provided another opportunity to contact residents and increase HCV screening. For example, demand for COVID-19, influenza, and pneumococcal vaccinations increased during the COVID-19 pandemic, and we also found that some residents who had never been to LDPHC appeared for the first time during this period. 

The HCV care cascade can be divided into several steps, including anti-HCV testing, HCV RNA testing, access to care, and DAA treatment [17]. Usually, barriers are found in the access to care and treatment access among people living in remote areas. In the present study, because most anti-HCV-seropositive patients were older adults who seldom saw a doctor in a tertiary care center, it was identified that transportation and the complex processes of tertiary referral centers were barriers that deterred patients in Liouguei District from seeking HCV treatment. Several strategies have been recommended in previous studies to overcome these barriers, including outreach hepatology clinics, decentralization, and task-shifting to non-specialists [18,19]. An outreach hepatology clinic can provide easy access to care and HCV treatment. However, it needs support from medical centers, which is usually a temporary method of support. In the newly developed collaborative model, with the aid of a shuttle bus, scheduled referral, and a simplified process in the hospital, patients can arrive at E-Da hospital easily and see physicians who are already familiar with the referral process. Peer effect is another advantage of the new collaborative model. Patients referred by LDPHC take the same bus to E-Da hospital and usually encourage each other, which increases patients’ acceptance of referral and treatment. Most barriers for referral were addressed and solved in our model, which was reflected in the high referral rate of our patients. However, 11.4% of LDPHC patients still refused referral. DAA treatment performed by general practitioners may overcome the barrier of access to care and is a good method by which to increase treatment uptake.

Although the Taiwan NHI authorized that DAAs can be prescribed by all physicians, including general practitioners, in October 2021, we still adopted the present collaborative model because of the following considerations. First, LDPHC patients who refused referral were influenced by lack of awareness, unwillingness, and health problems, which were difficult to be overcome using other strategies. Second, this referral model was developed by integrating the daily practice of two medical institutions without increasing the workload. The most important task of general practitioners in this model was to increase the number of patients willing to receive anti-HCV testing. Additional training may be needed if general practitioners want to perform DAA treatment, which may possibly become an implementation barrier. Third, this model provides sustained referral, and patients can be referred at any time. Such flexibility is especially important because HCV screening is performed continuously by LDPHC, and patients tend to accept referral when they are first informed of HCV infection. 

HCC is one of the late complications of HCV infection. Studies have shown that cirrhosis, increasing age, positive HCV RNA, elevated serum ALT levels, and HCV genotype 1 were associated with developing HCC [20,21]. LDPHC patients were at risk for HCC. In the present study, 4 out of 70 patients who were successfully referred to E-Da hospital were found to have HCC. Residents in Taiwan mountainous regions are relatively older and lack medical resources. The ages of these 4 patients ranged from 66 to 72 years, and all were HCV-viremic patients. In addition, none of these patients had received abdominal ultrasonography in the past 10 years. Although abdominal ultrasonography is not mandatory for HCV therapy, it should be considered in patients with higher risk of developing HCC. 

In addition to HCV treatment, residents in remote areas also have other healthcare needs—either diagnosis and treatment for chronic diseases such as heart disease or examinations such as esophagogastroduodenoscopy or colonoscopy. For example, the fecal immunochemical test (FIT) is used for colorectal cancer screening, and colonoscopy is recommended for people who test positive [22]. The present model can also be applied to referrals for colonoscopy. A total of 24 residents with positive FIT successfully received colonoscopy in E-Da hospital via the collaborative referral model during this period. Polypectomy was performed in 7 patients, and surgical intervention was performed in 1 patient with advanced colon cancer. Before we established and implemented this referral model, residents of the remote Liouguei District who had positive FIT were seldom referred for colonoscopy. Because of the success in LDPHC, we think this collaborative referral model can be applied to other primary clinics or public health centers in other remote regions, increasing the health of residents in these regions. 

The present study has several limitations. First, although we reached a highly successful referral rate and the overall screening coverage rate increased from 40% to 69.4% after referral, 30.6% of residents still did not receive anti-HCV testing. In addition, 13 out of 22 anti-HCV-seropositive patients who received anti-HCV testing at other hospitals still needed referrals, representing a lower rate of access to care from other hospitals. However, because LDPHC is currently the only clinic participating in this model, the screening coverage rate and referral rate can be expected to increase if this model can be expanded to other clinics in Liouguei District. Second, once-in-a-lifetime hepatitis B and C screening services in Taiwan were restricted to people between the ages of 45 and 79. Among residents older than 45 years in Liouguei District, 11.6% of residents are older than 80 years and are excluded from HCV screening services. Other methods are needed to include this population for HCV screening. In addition, although the prevalence of HCV infection is relatively low in the younger generation in Taiwan, DAA treatment for patients under 45 years old might have a greater impact to prevent the progression of chronic liver disease. Further cost-effectiveness evaluation of HCV screening for population under 45 years old is also warranted. 

## 5. Conclusions

The collaborative referral model described herein demonstrates a good model of HCV screening, access to care and treatment in a Taiwan mountainous region, even during the COVID-19 pandemic. In addition, sustained referral is possible based on this routine referral model, and the model has the potential to be applied for the referral of other diseases or examinations from remote regions.

## Figures and Tables

**Table 1:** Strategies to overcome barriers in HCV care cascades.

1. Integration of a team to strengthen collaboration between LDPHC and E-Da hospital. A team was established comprising LDPHC staff, hepatologists, and case managers from E-Da hospital. A consensus meeting was held before HCV screening and referral began. Online meetings were held as needed to overcome barriers.
2. Provision of HCV screening as part of integrated services at LDPHC. LDPHC staff educated residents who visited LDPHC for medical needs about the importance of HCV treatment and encouraged the residents to receive HCV screening, even when residents initially came to LDPHC for the COVID-19 vaccine. 3. A shuttle bus and scheduled referrals to increase referral acceptance. Direct transportation to E-Da hospital and appointment with a physician who was familiar with this model increased the convenience of referral, which improved patients’ accessibility. 4. A simplified process to reduce outpatient waiting time. A simplified process was built to reduce waiting time to see a doctor and to receive ultrasonography, which is especially important to those unfamiliar with the processes in a tertiary referral center. 5. Routine referral model. This model was developed through its integration into the daily practice of two medical institutions without increasing the workload. Therefore, it is a routine referral model that can work sustainably without interruption.

HCV, hepatitis C virus; LDPHC, Liouguei District Public Health Center.

**Table 2:** Characteristics of 70 HCV-infected subjects with successful referral.

Characteristics	Patients with Detectable HCV RNA	Patients without Detectable HCV RNA	*p* Value
	(*n* = 38)	(*n* = 32)	
Age, years	64.8 ± 8.1	64.0 ± 12.0	0.754
Male sex	20 (52.6)	11 (34.4)	0.126
HBV co-infection	3 (7.9)	4 (12.5)	0.695
Liver cirrhosis *	2 (5.3)	1 (3.1)	1.0
Hepatocellular carcinoma	4 (10.5)	0 (0)	0.122
AST, IU/L	70.6 ± 87.4	25.8 ± 11.2	0.005
ALT, IU/L	69.0 ± 79.8	24.0 ± 10.7	0.003
White cell count ×10^3^/μL	6.1 ± 2.4	7.2 ± 2.4	0.066
Hemoglobin, g/dL	13.8 ± 2.0	13.3 ± 1.9	0.315
Platelet count, ×10^3^/μL	208.3 ± 68.1	256.2 ± 91.0	0.016
Albumin, g/dL	4.2 ± 0.4	4.3 ± 0.4	0.179
Total bilirubin, mg/dL	0.7 ± 0.3	0.6 ± 0.4	0.291
Baseline HCVRNA, log IU	5.9 ± 1.0	-	-
HCV genotype,		-	-
1/2/3/6	15 (39.5)/15 (39.5)/ 1 (2.6)/6 (15.8)	-	-
1 + 2	1 (2.6)	-	-

* Liver cirrhosis was assessed by ultrasonography. Values expressed as mean ± standard deviation or sample size and proportion (%). HCV, hepatitis C virus; HBV, hepatitis B virus; AST, aspartate aminotransferase; ALT, alanine aminotransferase.

**Table 3:** Treatment outcomes of 35 patients receiving DAAs.

	*n*/N (%)
DAA regimens	
GLE/PIB	28/35 (80.0)
SOF/VEL	7/35 (20.0)
Complete treatment	33/35 (94.3)
EOTVR	33/35 (94.3)
SVR12 (ITT)	32/35 (91.4)
SVR12 (PP)	32/32 (100)
Explanation for non-SVR12	*n* = 3
Virological failure	0
Non-virological failure	
Death during treatment	1
Lost to follow-up	2

DAAs, direct-acting antivirals; GLE, glecaprevir; PIB, pibrentasvir; SOF, sofosbuvir; VEL, velpatasvir; EOTVR, end-of-treatment viral response; SVR, sustained virological response; ITT, intention-to-treat; PP, per-protocol.

## Data Availability

All the data were obtained upon the request to the corresponding authors.
